# Cognitive assistance for action selection: Challenges and approaches

**DOI:** 10.3389/fpsyg.2022.1031858

**Published:** 2023-01-04

**Authors:** Benjamin Strenge, Thomas Schack

**Affiliations:** Neurocognition and Action Research Group, Faculty of Psychology and Sports Science, Center for Cognitive Interaction Technology (CITEC), Bielefeld University, Bielefeld, Germany

**Keywords:** assistance systems, human augmentation, human enhancement, SDA-M, mental representation structures, sustainability

## Abstract

Cognitive assistance systems aim at compensating shortcomings of natural cognition concerning specific activities. Notable progress has been made regarding data acquisition, analysis, and the exploration of technical means for supporting human action selection and execution. The related challenges and potential solutions can be associated to four largely independent questions: What actions should be executed, when this must or should be done, whether assistance is needed for a specific action, and if so, how the action should be supported. A broad range of technological and methodical approaches can be taken for tackling each of these issues, including recent advances and new challenges in the automatized analysis of task-related mental representation structures.

## 1. Introduction

In the 2020s, pandemics, wars, and climate change pose enormous challenges to human civilization. At the same time, many people's daily lives are often dominated by less far-reaching but equally non-trivial questions such as: What food should I buy, and how and in what order can I then prepare the available ingredients in order to adhere to a particular diet plan and achieve the relevant environmental, health, and athletic goals? Other common types of issues at work and at home revolve around even more narrowly focused questions such as: How can I assemble the new piece of furniture as efficiently and safely as possible? In such situations, it would be desirable to always have an expert on hand to accompany you and help out with appropriate hints and advice—or an appropriately “intelligent" technical assistance system, such as the fictional Tony Stark's J. A. R. V. I. S., which compensates for shortcomings and error-proneness of human cognitive systems. Arguably, the current state of science and technology is still fairly far from this vision, and yet substantial progress has been made in relevant sub-areas in recent years. On this basis, we aim to classify in the following where we currently stand and which essential challenges still have to be solved from our perspective in the future in order to offer cognitive assistance suitable for everyday use through technical systems that leads to better selection and error-free, pleasant execution of human actions.

## 2. The four primary independent issues

In general, the challenges to be solved in the area of cognitive action assistance can be roughly assigned to answering four independent questions:

1. **What** actions should be executed?
2. **When** must or should this be done?
3. **Is assistance needed** for a specific action? And if so:
4. **How** should the action be supported?

**Figure 1:**
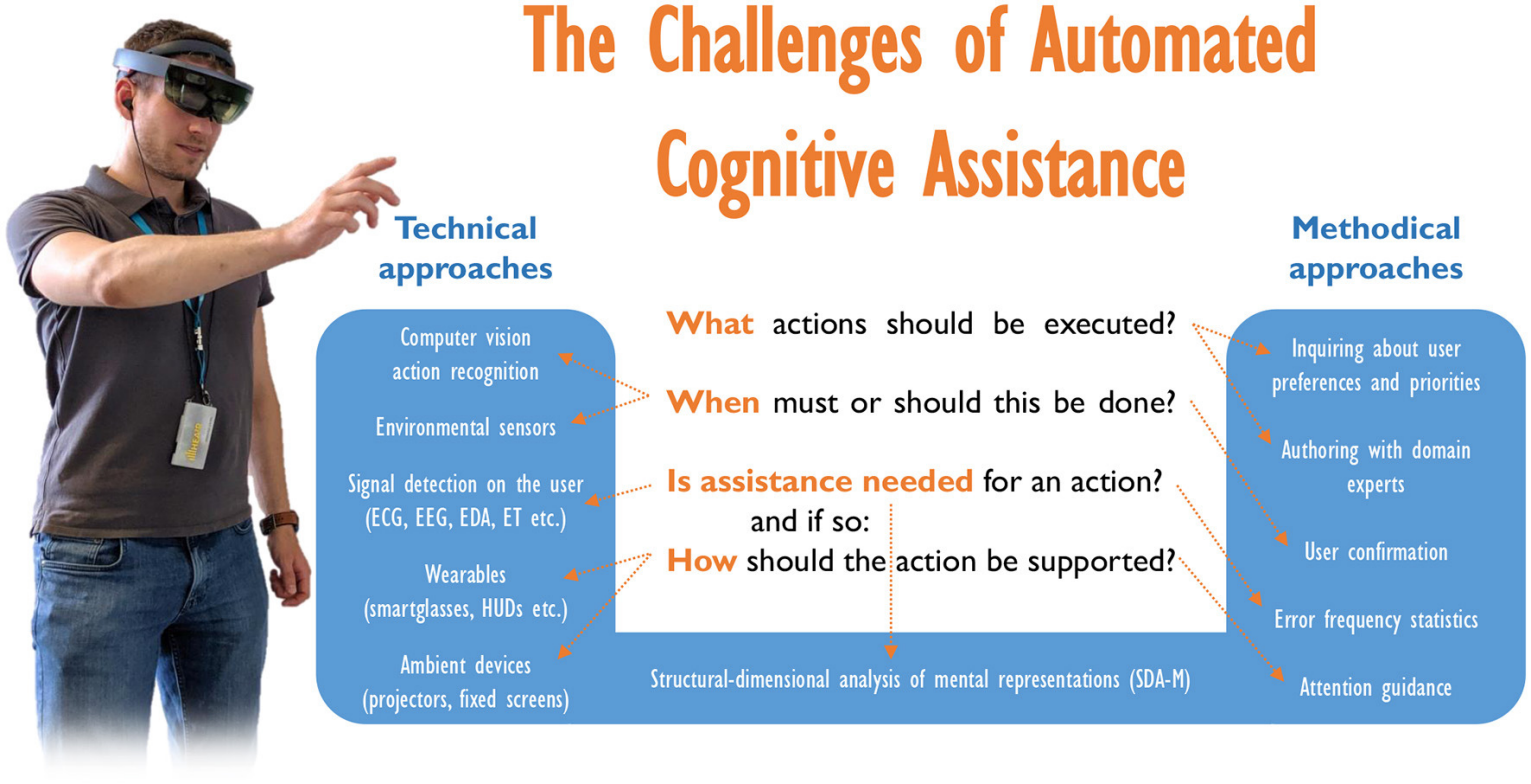
Fundamental issues and potential approaches to cognitive assistance for human action support.

Figure 1 provides an overview about these issues and potential approaches that have been investigated by different researchers over the past years.

By its very nature, the answer to question 1 (“*What actions should be executed?*”) is often highly context-dependent and individual. For example, the selection of "correct" actions in the area of nutrition is a highly complex, multi-layered issue that depends strongly on individual preferences, priorities and life circumstances (e.g., Franz et al., 2014; Cecil and Barton, 2020), and the relevant scientific body of knowledge is constantly evolving (Mozaffarian et al., 2018; Ridgway et al., 2019). In such cases, it is conceivable in principle that a technical system will inquire about the corresponding **preferences and priorities of the user** and then derive suitable suggestions for action on the basis of certain rules or heuristics. Depending on the complexity of the respective area, typically only a rough approximation to the theoretical optimum can be achieved. The assembly of a piece of furniture, on the other hand, can be specified largely independent of context as a simple sequence of action steps. For such activities, it is relatively easy and unambiguous to determine which action is necessary in which step. In any case, addressing question 1 commonly requires more or less extensive **authoring** for each activity to be supported, in the context of which the rules, criteria, or sequences are explicitly identified and formalized in cooperation with domain experts, and possibly a more or less extensive database must be made available to the system (e.g., the nutritional values of various ingredients and dishes, or the tools that can be used for certain assembly steps).

The treatment of question 2 (“*When must or should this be done?*”) could again be divided into a) the **determination of an optimal sequence** of different actions when performing several activities in parallel and b) the **recognition of the current state**. The search for an optimal temporal ordering of actions is addressed by theoretical and practical computer science in the context of scheduling algorithms under various conditions, but unfortunately many variants of this problem have turned out to be NP-complete, i.e., practically intractable (Ullman, 1975). Thus, in many cases, a technical assistance system can only find and propose approximately optimal solutions. Nevertheless, considering the properties and limitations of human working memory and cognitive bottlenecks in attention and executive functions (Anderson et al., 2004; Borst et al., 2010; Salvucci and Taatgen, 2010), any help in multitasking is likely to be welcome. Recognition of the current activity state is obviously necessary for a cognitive assistance system to know when to be proactive. In this respect, impressive progress has been observed for years in the fields of computer vision and **action recognition** facilitated by machine learning techniques (e.g., Baccouche et al., 2011; Schröder and Ritter, 2017; Abdulazeem et al., 2021), but overall these have so far typically still been limited to specific, well-defined applications and require prior recording of, or access to, huge amounts of data. For the foreseeable future, therefore, technical systems are likely to fall short of the power of human cognition in this area. A less elegant but technically simpler and much more robust approach is to ask for **users' conformation** that they executed an action or otherwise initiated or recognized a relevant change of the activity state. Other possibilities lie in the use of **environmental sensors** and other external data sources that can provide information on the current activity status. For example, if a user shall be assisted while operating a complex industrial machine, that machine may already be connected to suitable external or built-in sensors for gathering process status information and make them available to the assistance system.

The answer to question 3 (“*Is assistance needed for a specific action?*”) can be approached in a static or dynamic way, or by a combination of these two approaches. For the static estimation of whether assistance is needed for certain actions, on the one hand, **statistics on error frequency** or the generally expected need for assistance can be used if they are available. However, since task-related prior knowledge and relevant expertise can differ greatly between individuals, such approaches can only serve as very rough heuristics. In contrast, a more precise assessment can be obtained on the basis of an individual task-related **structural-dimensional analysis of mental representations** (SDA-M) (Schack, 2012), whose current status and perspectives are outlined in more detail in the following sections. Another, also complementary feasible way to find out when assistance is needed could be found in the **detection of signals on the user**, for example by means of portable electroencephalography (EEG), electrocardiography (ECG), and eye tracking (ET) systems, or by measuring electrodermal activity (EDA). During activity execution, confusion or a lack of crucial information can trigger an acute stress response, which can be measured, for example, as a reduced heart rate variability (e.g., Camm et al., 1996; Szakonyi et al., 2021), increased skin conductance (Critchley, 2002), or decreased pupil dilation (Henckens et al., 2009), thus providing an indication that assistance is needed.

The handling of the fourth and last question (“*How should the action be supported?*”) again depends strongly on the field of application and the complexity of the activities to be assisted. Portable devices are generally advantageous if the activity is not performed exclusively in a stationary position (e.g., sitting or standing at a fixed workspace). According to our perspective, wearables such as spatial computing **smart glasses**, which can augment reality by displaying arbitrary virtual elements and helpful instructions directly where the action needs to be performed in the real three-dimensional space, are particularly suitable to support a wide range of activities effectively and comprehensibly. But also simpler 2D head-up displays (HUDs), headsets, or "ambient devices" placed at fixed positions in the environment of the activity performance (e.g., using projectors) can offer assistance functions while users can freely perform the tasks without having to hold the assistance device in their hands. Complementary to the usual considerations concerning usability and user experience in the context of interaction design, an issue of particular importance for effective action assistance is proper attention guidance, especially when using wearable devices with limited fields of view (Renner and Pfeiffer, 2017a,b,c; Renner et al., 2018).

## 3. Recent advances and successes of SDA-M-based approaches

Structural-dimensional analysis of mental representations (SDA-M) is a method that originated in cognitive psychology and has later also been established in sports science, cognitive robotics, and human-technology interaction. It is based on the cognitive action architecture approach (CAA-A) by Schack (2004). The CAA-A postulates that the control of human movements is based on mental representation units, the so-called basic action concepts (BACs), and their structural composition in relation to one another (Schack and Frank, 2021). Within the hierarchical cognitive architecture of skilled action, the level of mental representations that uses BACs as a means is linked to the highest regulatory level of mental control, which intentionally controls overarching strategies, as well as to lower levels of sensorimotor representation and control that utilize and automatize functional systems and basic reflexes. Accordingly, BACs connect goal-directed functional and perceptual aspects of actions to sensory effects of movements. The individual strengths of associations between BACs of an activity in long-term memory can be analyzed with SDA-M software tools based on a special semi-automatic survey procedure (the so-called "split procedure"). These data can then be visualized *via* hierarchical clustering algorithms in the form of dendrograms to allow appropriately trained experts to assess the mental representational structure and **identify expectable problems in action execution** (e.g., Heinen et al., 2002; Schack, 2004; Schack and Hackfort, 2007; Vogel, 2016). In recent years, this procedure has been advanced for use in the cognitive assistance systems ADAMAAS (Essig et al., 2016) and AVIKOM (Neumann et al., 2020) by automating the diagnosis step. For this purpose, the Correct Action Selection Probability Analysis (CASPA) algorithm has been created, which is based on approaches from the cognitive architecture ACT-R by Anderson et al. (2004) and estimates for each individual action from a sequence of actions the individual probability whether a user will be able to select a correct subsequent action after completing the action on his/her own, or will need assistance in doing so (Strenge et al., 2019). Empirical studies indicate that the majority of all action errors that actually occurred could be correctly predicted in this way (Strenge et al., 2020; Strenge and Schack, 2021). Cognitive assistance systems could use this information to proactively **prevent human action errors** in many cases through timely intervention and appropriate support.

## 4. Specific challenges of SDA-M-based approaches

A fundamental limitation regarding the applicability of current SDA-M-based approaches for cognitive assistance is that the prediction of error probabilities is currently **only possible for predefined action sequences** that satisfy some additional criteria (for details see Strenge et al., 2019). This is less problematic in many application domains than it might seem at first glance, because, as Sun (2004, p. 345) noted, "human everyday activities are mostly sequential." Another issue in practical use is the **time required for data collection** (the "split procedure") by users, since this increases quadratically with the incorporated number of mental representation elements (e.g., actions of an action sequence). Ongoing research aims to investigate whether this issue can be mitigated by sampling from a limited subsequence of actions and using this sample to derive an estimate of an individual's general task-related expertise. Furthermore, it is so far largely unclear how stable the captured mental representational structures are over time. Learning processes induced by practice lead to **changes in mental representational structures** such that the previously recorded SDA-M data no longer reflect the current state (Frank et al., 2013, 2016; Schack et al., 2014). Therefore, adequate test periods must be defined to measure and reflect task-relevant learning periods in order to always have sufficiently up-to-date information for meaningful cognitive assistance. Conversely, a dynamic adjustment of the extent of assistance to promote learning processes in line with the principle of learning facilitation in ISO 9241-110 is certainly desirable. Neumann et al. (2021) developed experimental approaches for tackling this issue.

## 5. Discussion

Overall, this perspective on which current issues concerning cognitive assistance systems are especially important, as well as the entailed considerations, should be regarded as a mostly subjective one that was derived to a large extent from research results and lessons learned in the context of two research projects on mobile cognitive assistance systems funded by the German Ministry of Education and Research (BMBF): Project ADAMAAS, which was conducted from 2015 to 2018, and project AVIKOM that started in 2019 and was scheduled to finish by the end of 2022. However, the scope and focus of these projects was narrower than what has been addressed here. Most of these further aspects could be related to what had been considered as “nice-to-have” functionality that did not make it into the research prototypes, or visions for the near future conceived by fellow researchers and partner companies. Future cognitive assistance systems may embrace these visions and solve the connected challenges or explore completely different innovative ways to support human activities and lead to better action selection. Regardless of the technological and methodological tools, it is hoped that sustainable and thriving future assistance systems will not only help out with limited, short-lived everyday problems, but also help their users, perhaps indirectly and subliminally, by choosing appropriate actions, to contribute to overcoming the great challenges of our time—the sustainable preservation of a habitable planet and functioning social structures.

## Data availability statement

The original contributions presented in the study are included in the article/supplementary material, further inquiries can be directed to the corresponding author.

## Ethics statement

Written informed consent was obtained from the individual(s) for the publication of any identifiable images or data included in this article.

## Author contributions

BS wrote the primary draft of the manuscript. All authors contributed to manuscript revision, read, and approved the submitted version.
